# Systemic Lupus Erythematosus and Vitamin D Deficiency Are Associated with Shorter Telomere Length among African Americans: A Case-Control Study

**DOI:** 10.1371/journal.pone.0063725

**Published:** 2013-05-20

**Authors:** Brett M. Hoffecker, Laura M. Raffield, Diane L. Kamen, Tamara K. Nowling

**Affiliations:** 1 Department of Medicine, Medical University of South Carolina, Charleston, South Carolina, United States of America; 2 Medical Research Service, Ralph H. Johnson Veterans Affairs Medical Center, Charleston, South Carolina, United States of America; University of Michigan, United States of America

## Abstract

Systemic lupus erythematosus (SLE) is a chronic systemic autoimmune disease that disproportionately affects African American females. The causes of SLE are unknown but postulated to be a combination of genetic predisposition and environmental triggers. Vitamin D deficiency is one of the possible environmental triggers. In this study we evaluated relationships between vitamin D status, cellular aging (telomere length) and anti-telomere antibodies among African American Gullah women with SLE. The study population included African American female SLE patients and unaffected controls from the Sea Island region of South Carolina. Serum 25-hydroxyvitamin D levels were measured using a nonchromatographic radioimmunoassay. Telomere length was measured in genomic DNA of peripheral blood mononuclear cells (PBMCs) by monochrome multiplex quantitative PCR. Anti-telomere antibody levels were measured by enzyme-linked immunosorbent assay (ELISA). Patients with SLE had significantly shorter telomeres and higher anti-telomere antibody titers compared to age- and gender-matched unaffected controls. There was a positive correlation between anti-telomere antibody levels and disease activity among patients and a significant correlation of shorter telomeres with lower 25-hydroxyvitamin D levels in both patients and controls. In follow-up examination of a subset of the patients, the patients who remained vitamin D deficient tended to have shorter telomeres than those patients whose 25-hydroxyvitamin D levels were repleted. Increasing 25-hydroxyvitamin D levels in African American patients with SLE may be beneficial in maintaining telomere length and preventing cellular aging. Moreover, anti-telomere antibody levels may be a promising biomarker of SLE status and disease activity.

## Introduction

Systemic lupus erythematosus (SLE) is a chronic autoimmune disease with diverse manifestations, with the presence of autoantibodies a unifying feature among patients. SLE often affects multiple organ systems, including but not limited to the skin, musculoskeletal, cardiovascular, renal, pulmonary, gastrointestinal, neuropsychiatric, and hematologic systems. [1] SLE occurs primarily in women (9∶1 ratio of women to men) during the reproductive years. SLE disproportionately affects African Americans and Hispanics with African Americans also having higher disease activity, rates of renal involvement, damage accrual and mortality compared to other ethnicities in the United States. [2], [3] Genetics play a role in the pathogenesis of SLE, but genetics alone are not sufficient for developing SLE, suggesting the influence of environmental triggers of disease expression.

Vitamin D deficiency is a potential environmental trigger of SLE and/or SLE-related disease activity. [4] Vitamin D is an essential steroid hormone with well-established effects on mineral metabolism, skeletal health, and more recently described effects on cardiovascular and immune health.[5]–[7] Mounting evidence has revealed that vitamin D deficiency contributes to the morbidity and mortality of multiple chronic diseases. [8] Both vitamin D2 and D3 are converted to 25-hydroxyvitamin D_3_ (25(OH)D), an inactive circulating form that must be hydroxylated to 1,25-hydroxyvitamin D_3_ (1,25(OH)_2_D) to be biologically active. However, determination of serum 25(OH)D levels is considered the best measure of vitamin D status. [9] Lifestyle factors have led to an increased prevalence of vitamin D deficiency in the general population, while improved availability and reliability of the serum 25(OH)D test have led to better awareness of the widespread deficiency. Because patients with SLE avoid the sun, a common trigger of disease flares, the risk of vitamin D deficiency is even higher among SLE patients than in the general population, particularly African Americans with SLE whose dermal pigmentation impedes conversion of vitamin D. [4] Vitamin D deficiency was associated with increased antinuclear antibody (ANA) positivity among healthy controls and increased B cell activation among patients with SLE. [10] Previously, we demonstrated a negative correlation between 25(OH)D and disease activity (SLEDAI score) among an African American Gullah cohort (SLE in Gullah Health or SLEIGH), a correlation which subsequently was demonstrated in many other SLE populations worldwide. [11].

Telomeres are DNA-protein complexes composed of short, tandem hexanucleotide repeats located at the ends of linear chromosomes on most somatic cells. The telomere end forms a loop, which requires a length of single-stranded 3′ overhang. [12] Telomerase, a telomere-lengthening enzyme, helps to maintain the 3′ overhang and thus the integrity of the chromosome. [13] Telomeres shorten during each cell division in the absence of telomere synthesis mechanisms such as telomerase. When telomeres reach a critically shortened length, chromosomal aberrations such as end-to-end fusion occur, and cells enter senescence or undergo apoptosis and are no longer able to replicate. Previous studies demonstrated accelerated telomere shortening in various circulating cells in SLE patients.[14]–[18] A relationship between leukocyte telomere length and vitamin D status was demonstrated in a cross-sectional measurement of leukocyte telomere length and 25(OH)D level among women from a large population-based cohort of twins in the United Kingdom. This study demonstrated that serum 25(OH)D levels were positively correlated with telomere length and this relationship remained significant after adjustment for age and other covariates. [19].

Anti-telomere antibodies are a type of anti-double stranded DNA antibody that specifically targets the hexanucleotide repeat sequences of telomeric DNA. SLE patients exhibit higher levels of anti-telomere antibodies compared to unrelated controls, and assays for anti-telomere antibodies are more specific for SLE compared to other autoimmune diseases. [20], [21].

To our knowledge, relationships between vitamin D status, telomere length and anti-telomere antibody positivity in SLE have not been investigated previously, particularly among African Americans, who are disproportionately affected by both SLE and vitamin D deficiency. In this study we analyzed the relationship between serum 25(OH)D levels, telomere length and anti-telomere antibody levels in a genetically similar population of African American Gullah women with SLE and unaffected controls. We demonstrate that SLE patients have shorter telomeres and higher anti-telomere antibody levels than age- and gender-matched controls. We further demonstrate an association between vitamin D deficiency and shorter telomere length and a positive correlation between anti-telomere antibodies and disease activity. Our results support the concept that increasing 25(OH)D levels may be beneficial in slowing or preventing premature cellular aging and that anti-telomere antibody measures serve as an important biomarker of SLE.

## Materials and Methods

### Ethics Statement and Study Subjects

This research was carried out with the approval of the Institutional Review Board at the Medical University of South Carolina. Written informed consent was obtained from all study participants. This case-control study was nested in a longitudinal observational cohort called SLE in Gullah Health (SLEIGH), which was started in 2002. A more complete description of the cohort was previously reported. [22] Briefly, eligible cases were: 1) age two years and above, 2) self-identified as African American "Gullah" from the Sea Island region of South Carolina, 3) diagnosed with SLE by meeting at least four of the 11 classification criteria as designated by the ACR, [23], [24] 4) able to speak and understand English, and 5) able and willing to give informed consent.

Stored DNA and serum from patients with SLE and age- and gender-matched controls enrolled in the SLEIGH Study was utilized for this study. Fifty-nine patients with SLE and fifty-nine age- and gender-matched unrelated controls (all African American Gullah) were included in the study. Patients were age-matched to their controls within two years, and all patients and controls were female. Twenty-nine of the patients with SLE had two samples taken at least three months apart, allowing us to analyze changes in 25(OH)D levels, telomere length and anti-telomere antibody levels over time.

### Clinical Disease Measurements

Disease activity was ascertained with the Systemic Lupus Erythematosus Disease Activity Index (SLEDAI) using medical records, laboratory testing and an in-person interview and physical examination. [25] Disease damage was ascertained using the Systemic Lupus International Collaborative Clinics (SLICC)/ACR damage index (SDI). [26] Assays for antinuclear antibodies (ANA) were performed by indirect immunofluorescence (IF) with HEp-2 cells (INOVA Diagnostics, Inc., San Diego, CA, USA). Detection of ANA at a dilution of 1∶40 was considered a minimal positive result. Anti-double-stranded DNA (dsDNA) antibodies were screened with a Crithidia assay (INOVA Diagnostics, Inc., San Diego, CA, USA). These assays were all performed in the clinical immunology laboratory at the Oklahoma Medical Research Foundation.

### Vitamin D Measurements

Baseline 25(OH)D levels of patients with SLE and age- and gender-matched controls from the SLEIGH cohort were measured in the laboratory of Dr. Bruce Hollis, a contract facility for the NIH for measures of vitamin D metabolites using a nonchromatographic radioimmunoassay (RIA). [27] Baseline levels of 25(OH)D are categorically defined as <10 ng/ml (severe deficiency), 10–19 ng/ml (moderate deficiency), 20–30 ng/ml (insufficiency), or >30 ng/ml (sufficient).

### Telomere Length Determination

Genomic DNA (gDNA) was extracted from PBMCs in blood samples collected from subjects, and telomere length was measured by monochrome multiplex quantitative PCR (MMqPCR) essentially as described. [28], [29] Adaptations to the protocol included the use of the SYBR green kit and the Lightcycler from Roche Diagnostics (Indianapolis, IN). The housekeeping single-copy gene albumin served as the normalizing gene. Melting curve analyses demonstrated well-separated single peaks for the telomere product and the single-copy gene product. gDNA isolated from a single preparation from the Jurkat T cell line served as a standard on each plate. Two standard curves were generated following PCR, one for the telomere signal and one for the single-copy gene signal. The average ratio of telomere signal to single-copy signal is proportional to the average telomere length per cell. All samples were run in triplicate within a plate and on two separate plates with similar results.

### Anti-telomere Antibody Measurements

Anti-telomere antibody levels for patient and control serum samples were measured using an anti-telomere IgG antibody ELISA kit (Biohit, Finland) following the manufacturer’s instructions. Serum samples were diluted 1∶100 and tested in duplicate and were run at least twice with similar results. The initial incubation time for the diluted serum on the pre-coated 96 well plate was increased to one hour to facilitate binding. Absorbance readings were measured with an ELx800 BioTek plate reader and converted to anti-telomere antibody concentrations by comparison to the provided standard at a concentration of 228 IU/mL. A positive result for anti-telomere antibodies was considered to be above 64 IU/mL, the 98^th^ percentile determined in healthy controls. [21].

### Statistics

To compare features between patients and controls, descriptive statistics were calculated using the Chi square distribution for proportions and t-tests for continuous variables. For data non-normally distributed, appropriate transformations were performed. Serum 25(OH)D level at baseline was examined as a continuous variable in relationship to the telomere length shortening rate between baseline and follow-up visits and in relationship to baseline telomere length, while adjusting for potential confounding variables such as age and disease duration. Spearman correlation coefficients were also determined. All statistical hypothesis tests were conducted at the Type I error level of 0.05.

## Results

**Figure 1:**
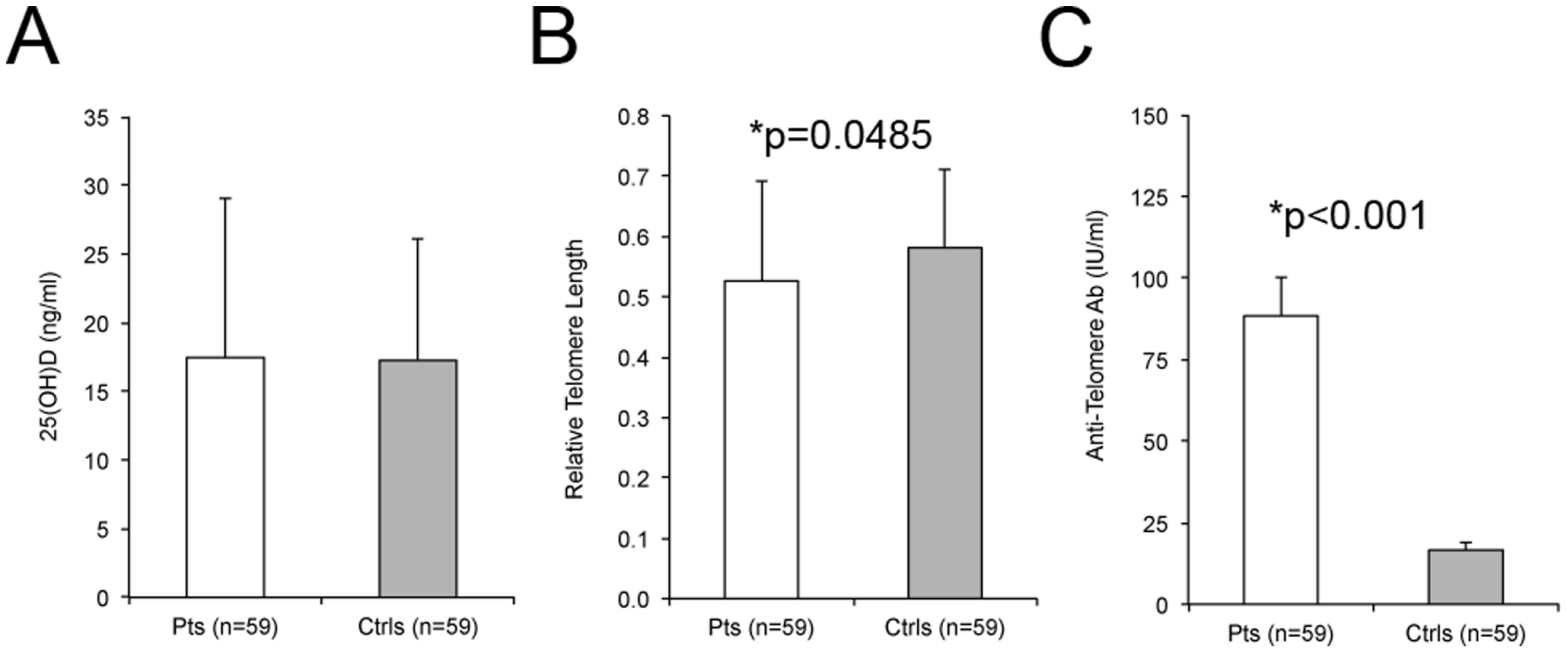
Vitamin D, telomere length and anti-telomere antibodies in SLEIGH patients and controls. A) 25(OH)D levels. B) Telomere length analysis. C) Anti-telomere antibody levels. Error, standard deviation by two-sample T-test with equal variance.

In this study, we analyzed serum and DNA from patients with SLE and unrelated controls from the SLE in Gullah Health (SLEIGH) cohort described in the Materials and Methods. Baseline demographic and clinical characteristics for the 59 patients and 59 controls are shown in Table 1. For the 29 patients with SLE who had both baseline and follow-up measurements analyzed, there was an average of 2.8±1.9 years between visits. On average, both the patients (17.5±1.5 ng/ml) and controls (17.3±1.1 ng/ml) analyzed in this study had 25(OH)D levels less than 20 ng/mL, and all but 10 subjects had levels less than 30 ng/ml (Fig. 1A).

**Table 1:** Demographic characteristics of the patients with SLE and unaffected controls and clinical characteristics of the patients with SLE.

	Gullah Controls (n = 59)	Gullah Patients with SLE (n = 59)
Age, mean ± SD (years)	39.86+11.57	39.88±11.63
Disease duration, mean ± SD (years)	n/a	7.4±6.7
% of patients with ACR criteria for renal disorder	n/a	55.9
% of patients with damage (SDI >1)	n/a	50.1
% with ESRD	0.0	13.6
% with ANA positive (>1∶40)	30.5	94.9
% with dsDNA positive	0.0	30.5
% with anti-telomere positive	1.7	45.8

For the 29 patients with 2 visits, the age used is from their baseline visit indicated above.

To determine whether differences in cellular aging exist in this population, telomere length was measured in circulating PBMCs. As expected, there was a significant inverse correlation between age and telomere length (p = 0.031, rho = −0.2); higher age predicts a shorter telomere length (p = 0.04). SLE patients had significantly shorter telomeres (0.526±0.022) than age- and gender-matched controls (0.581±0.017) (Fig. 1B, p = 0.0485) indicating that SLE patients in this cohort exhibit accelerated cellular aging.

Anti-telomere antibodies, a specific anti-dsDNA antibody that targets the telomeric ends of chromosomes, were shown previously to be elevated in lupus patients and be more specific for lupus compared to other autoimmune diseases in a largely Caucasian population. [21] Similarly, we observed that this cohort of African American SLE patients in our study had significantly higher levels of anti-telomere antibodies (88.4±11.7 IU/ml, ranging from 0 to 447 IU/ml) compared to age- and gender-matched controls (16.7±2.1 IU/ml, ranging from 0 to 80 IU/ml) (Fig. 1C, p<0.001). Individuals are considered positive for anti-telomere antibodies if their levels are 64 IU/ml or above. Among patients, there was a positive relationship between anti-telomere antibody levels and anti-dsDNA antibody levels (p<0.001, rho = 0.62). Interestingly, only 18 of the 59 patients were positive for anti-dsDNA antibodies while 27 of 59 patients were positive for anti-telomere antibodies. Of the controls, none were positive for anti-dsDNA and only one was positive for anti-telomere antibodies. This supports the idea that measuring levels of anti-telomere antibodies may be a more sensitive measure of SLE disease activity than anti-dsDNA antibodies.

### Relationship between Vitamin D Levels, Telomere Length and Anti-telomere Antibodies

**Figure 2:**
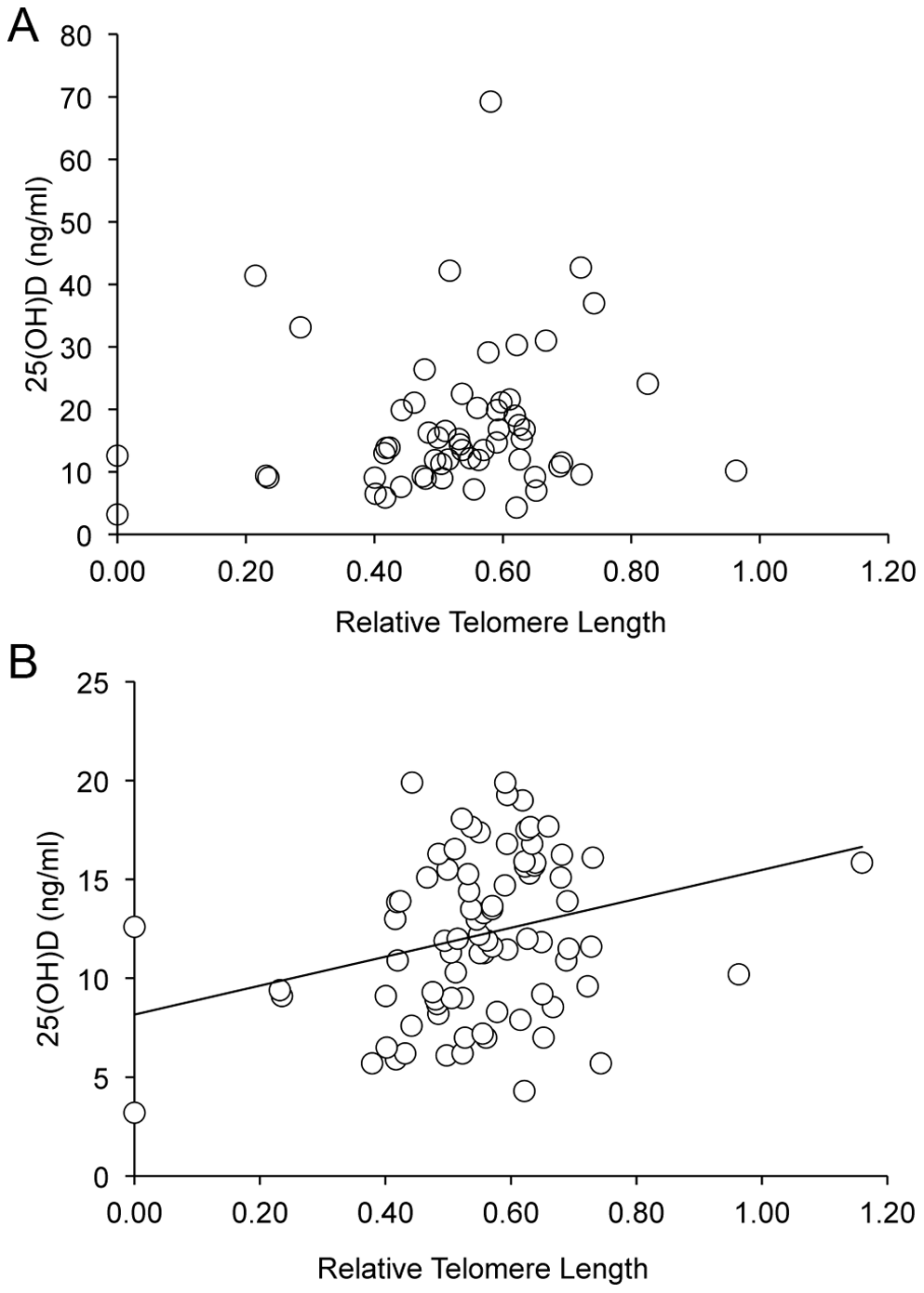
Vitamin D levels are positively correlated with telomere length in SLEIGH subjects. A) Correlation between 25(OH)D levels and telomere length in SLEIGH patients (n = 59) and B) all SLEIGH subjects (both patients and controls with 25(OH)D levels less than 20 ng/ml) (n = 84).

We next analyzed the relationship between 25(OH)D levels and telomere length. No correlation between 25(OH)D levels and telomere length was observed among the patients (Fig. 2A; p = 0.0858, rho = 0.23) or controls (p = 0.9867, rho = 0.002). Among patients with the highest disease activity (SLEDAI score ≥6), there was also no correlation between 25(OH)D levels and telomere length (p = 0.0950, rho = 0.45).

The prevalence of vitamin D insufficiency was high among both patients and controls in this cohort. Therefore, we tested for an association between 25(OH)D levels and telomere length in all subjects with 25(OH)D levels <20 ng/ml and observed a statistically significant correlation between short telomeres and low 25(OH)D levels (Fig. 2B; p = 0.0102, rho = 0.28).

We then tested for correlations between 25(OH)D and anti-telomere antibody levels. No correlation was observed between 25(OH)D levels and anti-telomere antibody levels for either patients or controls. However, there was a trend towards an inverse relationship between having a short telomere (≤0.56) and anti-telomere antibody level when analyzing all subjects (p = 0.055). This trend was also observed between short telomeres and anti-dsDNA antibody levels, although neither trend reached statistical significance.

### Relationship between Disease Parameters and Telomere Length or Anti-telomere Antibodies

**Figure 3:**
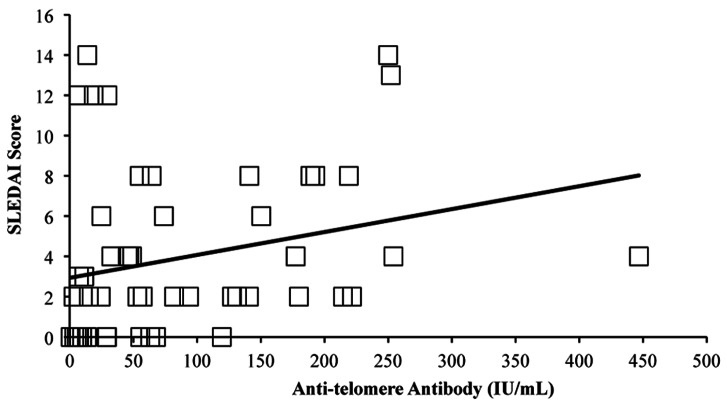
Anti-telomere antibody levels are positively correlated with SLEDAI score in SLEIGH patients. SLEDAI scores available for SLE patients at their baseline visit were compared to their anti-telomere antibody levels at their baseline visit (n = 52) by Spearman’s rank test.

Correlations between telomere length and damage (SDI) score, presence of end stage renal disease (ESRD), total ACR criteria count and disease activity (SLEDAI) score within ten days of visit were analyzed. No significant correlations were observed. There also were no correlations between anti-telomere antibody levels and SDI score or between anti-telomere positivity and high/low SDI damage. However, there was a significant relationship between anti-telomere antibody level and SLEDAI score in patients (Fig. 3; n = 52, p = 0.009, rho = 0.36). Anti-dsDNA levels also correlated with SLEDAI score to a similar degree as the anti-telomere antibody levels (p = 0.012, rho = 0.35).

### Relationship between Increase in Vitamin D, Telomere Length and Anti-telomere Antibody Levels Over Time

**Figure 4:**
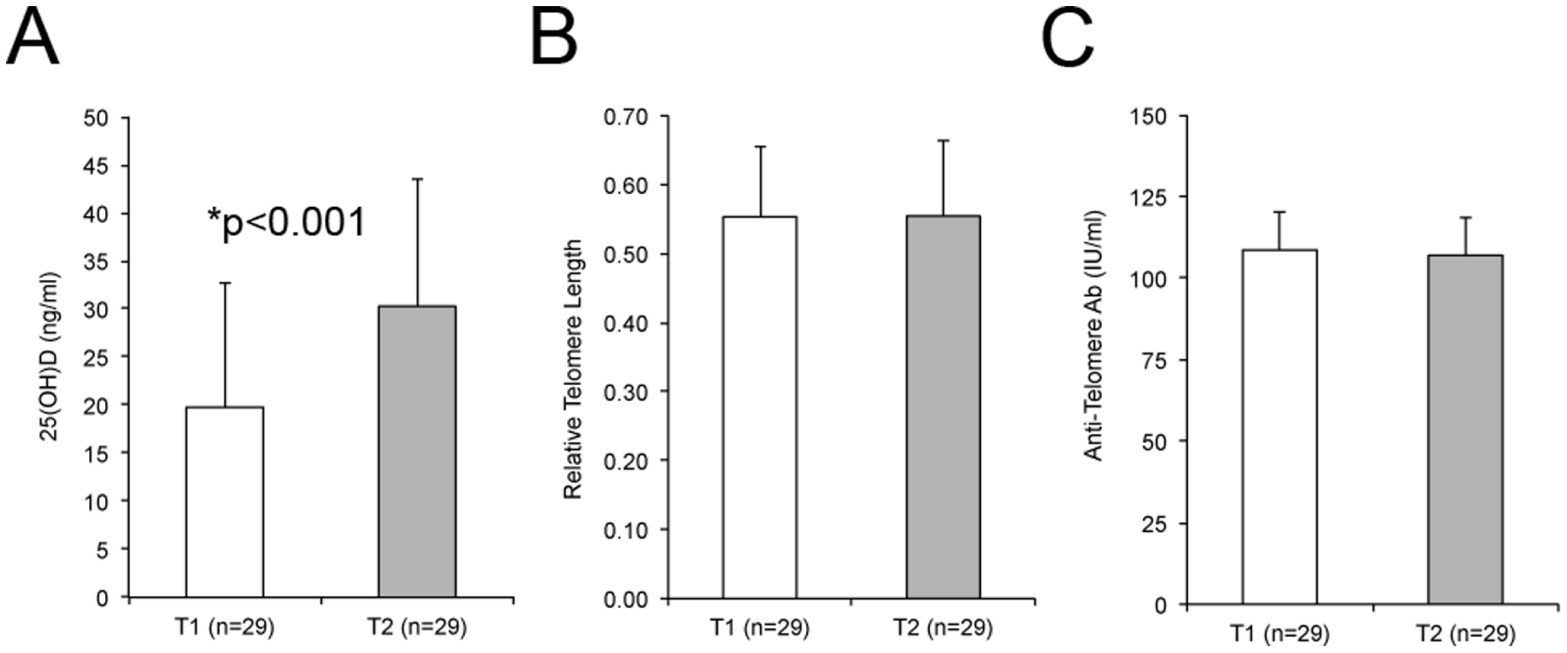
SLEIGH patients had a significant increase in vitamin D levels, but not in telomere length over time. Vitamin D levels, telomere length and anti-telomere antibody levels were measured in 29 of the 51 SLEIGH patients in Fig. 1 at a follow-up visit and compared to their levels at their baseline visit. A) 25(OH)D levels. B) Telomere length analysis. C) Anti-telomere antibody levels. T1, baseline visit; T2 is follow-up visit for patients in T1.

Nearly all of the patients in the cohort had insufficient or deficient levels of 25(OH)D. To determine whether increasing 25(OH)D levels over time would impact telomere length and/or anti-telomere antibody levels, 29 of the 59 patients in the cohort were followed over an average period of 2.8±1.9 years. The average 25(OH)D levels in these 29 patients significantly increased between the first (T1 = 19.8±1.7 ng/ml) and second visits (T2 = 30.3±1.7 ng/ml) (Fig. 4A, p = 0.0004), which was likely due largely to increased awareness of vitamin D status and starting supplementation. However, there was no significant change in average telomere length (Fig. 4B; T1 = 0.554±0.013, T2 = 0.554±0.014) or in average anti-telomere antibody levels (Fig. 4C; T1 = 108.6±11.6 IU/ml, T2 = 107.0±11.9 IU/ml). In analyzing the relationship between 25(OH)D levels and telomere length and/or anti-telomere antibodies in the longitudinal patients, no correlations were observed between change in 25(OH)D levels and telomere length over time or change in 25(OH)D levels and anti-telomere antibody levels over time. Additionally, no relationship was observed between telomere length and total SDI score, ESRD, total ACR criteria count or SLEDAI score at follow up in the longitudinal patients.

Interestingly, when categorizing the 29 longitudinal patients as 25(OH)D insufficient/deficient (<30 ng/ml) or 25(OH)D sufficient (≥30 ng/ml) at follow up visit, we observed that the 25(OH)D sufficient patients tend to have longer telomeres, although the difference was not statistically significant (Table 2).

**Table 2:** Telomere length and anti-telomere antibody levels in longitudinal SLE patients with sufficient compared to insufficient vitamin D levels.

25(OH)D	25(OH)D Average(ng/ml)	Telomere Length Average	Anti-Telomere Ab Average (IU/ml)
Insufficient(<30 ng/ml)n = 18	21.75±5.83	0.54±0.08(33% “long”)	96.88±76.71(56% positive)
Sufficient(≥30 ng/ml)n = 11	41.61±11.18	0.58±0.14(55% “long”)	122.56±112.38(55% positive)
Insufficient vs. Sufficient p-value	<0.01	0.33	0.47

Telomere length is considered “long” at a cut-off of >0.56. Anti-telomere antibodies are considered “positive” at a cut-off of 64 IU/mL.

## Discussion

In SLE, vitamin D deficiency is thought to act as a possible trigger of disease and/or disease activity, and cellular aging/senescence may contribute to disease progression. Here we explored a potential relationship between vitamin D, telomere length and anti-telomere antibodies in Gullah African American women with SLE to determine whether 25(OH)D levels may impact telomere length and cellular aging in SLE. 25(OH)D levels, telomere length and anti-telomere antibody levels were measured in the patients and age-matched unaffected control subjects. One strength of this study lies in the study population. The Sea Island Gullah African American population enrolled in the SLE in Gullah Health (SLEIGH) study, an established cohort of African American patients with SLE, is an ideal cohort for defining genetic and environmental risk factors for SLE because of their genetic homogeneity with very low non-African genetic admixture and preserved family unit. [22] The strong influence of ethnicity on overall and renal outcomes in SLE may reflect varying environmental exposures and/or genetic factors. Similar disparities are seen in levels of 25(OH)D, with African Americans having a disproportionately higher prevalence of vitamin D deficiency. [30] African Americans also have a three-fold increased incidence of SLE, develop SLE at an earlier age, and have increased morbidity and mortality compared with Caucasians. [2], [3] The low genetic admixture and the high incidence of SLE in the Gullah population lends itself towards an analysis that is less likely to be hampered by variability due to wide genetic differences.

We observed that both SLE patients and unaffected controls in this Gullah cohort were largely vitamin D deficient at their baseline visit similar to previous studies in other populations [4], [31]. Vitamin D has a variety of immunomodulatory functions, [10], [32], [33] and may be a contributing factor to various diseases and overall mortality. [8] Vitamin D has autocrine and paracrine roles as it affects T cells, B cells and dendritic cells, which all express the vitamin D receptor (VDR) and the vitamin D activating enzymes necessary to produce the active form of vitamin D, 1,25(OH)_2_D. The VDR gene encodes a transcription factor that is activated by 1,25(OH)_2_D and modulates many biologic processes. The overall immunologic effects of 1,25(OH)_2_D include downregulating Th1 immune responses, modulating the differentiation of dendritic cells (DCs), and lowering proliferation of activated B cells, while upregulating regulatory T cells and preserving innate immune responses.[32]–[34] In particular, 1,25(OH)_2_D inhibits type 1 interferon (IFN), which plays an important role in SLE. [35].

In measuring telomere length, we first tested whether telomere length in PBMCs is representative of what is occurring in a specific cell type such as T cells. We compared telomere lengths in total PBMCs to telomere length in T cells isolated from the same blood sample of nine lupus patients. Results from these nine patients indicated that, on average, the telomere length in PBMCs of lupus patients is similar to that observed in their circulating T cells (data not shown). Therefore, we determined that telomere length in PBMCs of this cohort would provide a representative assessment of differences in telomere length. We observed that SLE patients have significantly shorter telomeres than their age- and gender-matched controls. As expected, telomere length was inversely correlated with age and a higher baseline age was predictive of a shorter telomere.

Influences causing telomeric loss include genetic and epigenetic factors, sex hormones, reactive oxygen species, defects in telomere repair, inflammatory reactions and increased cellular turnover.[36]–[38] Immune system function is highly dependent on the maintenance of telomere length. Telomere shortening occurs naturally over time in T and B lymphocytes, granulocytes, monocytes, and natural killer cell populations. This leads to a decrease in adaptive immunity and may cause predisposition to autoimmune responses, increasing susceptibility for chronic inflammatory diseases. [36], [37], [39] Leukocytes have the unique ability to activate telomerase when stimulated and delay the loss of telomeric DNA enhancing their function and immune response. [40] However, in situations where the immune system is constantly stimulated an increase in senescent cells and telomere shortening occurs. [41] This was observed in several immune cell types in lupus where chronic endogenous cell activation and inflammation occurs. Specifically, telomeres in mononuclear cells (MNCs) and polymorphonuclear neutrophils (PMNs) were demonstrated to decrease normally with age, but patients with SLE displayed premature and accelerated telomere shortening in these cells. [16] In other studies, patients with SLE showed accelerated telomere shortening and higher levels of senescent telomeric DNA in PBMCs, leukocytes or T cells compared to age- and gender-matched controls. [14], [17], [18].

Given the immunomodulatory effects of vitamin D and that telomeres exhibit premature shortening in immune cells of patients with SLE, we analyzed potential relationships between 25(OH)D levels and telomere length in PBMCs in the Gullah cohort. Although 25(OH)D levels did not correlate with telomere length per se in either the patients or the control subjects, there was a statistically significant correlation of shorter telomeres among those with 25(OH)D deficiency (<20 ng/ml) when analyzing all subjects at baseline visit. There also was a trend towards an inverse relationship between short telomeres and anti-telomere antibody level. When examining 25(OH)D levels and telomere length in a subset of the patients over time there was an increase in 25(OH)D levels (likely due to initiation of vitamin D supplements after discovery of the low 25(OH)D at baseline), but no significant change in telomere length and no correlation between 25(OH)D levels and telomere length at follow-up. However, we did observe that patients that remained at insufficient/deficient levels of 25(OH)D (<30 ng/ml) at follow-up had shorter telomeres than those patients that had sufficient levels of 25(OH)D (>30 ng/ml). We speculate that repleting 25(OH)D levels in these patients may prevent or slow the premature shortening of their telomeres to a rate equivalent to the normal aging process. A longer follow-up period in the longitudinal study is needed to provide confirmation of this initial observation.

As mentioned previously, one hallmark of SLE is the expression of anti-dsDNA antibodies, often before overt signs or symptoms of disease. Anti-telomere antibodies, a type of anti-dsDNA antibody that specifically target telomeres, are present in SLE and appear to be more specific for SLE than anti-dsDNA antibody tests. [20], [21] We postulated that targeting of the telomeres by anti-telomere antibodies may impact the accelerated shortening of telomeres observed in SLE patients. Therefore, we measured anti-telomere antibody levels in the Gullah cohort and tested for relationships between anti-telomere antibody level and telomere length. Our data, in agreement with previously published data, demonstrated that SLE patients have significantly higher levels of anti-telomere antibodies compared to age- and gender-matched unaffected controls. In fact, more SLE patients were positive for anti-telomere antibodies than for anti-dsDNA antibodies. These results support the suggestion that anti-telomere antibodies may serve as an important biomarker for characterizing disease activity independent of anti-dsDNA antibodies, in addition to the high disease specificity that was observed in previous study populations. [20], [21] Importantly, our results are the first to demonstrate a strong relationship between anti-telomere antibody levels and disease activity in African American SLE patients.

No significant correlation between anti-telomere antibody level and telomere length among patients was observed when analyzing absolute values or degree of change over time. We also analyzed anti-telomere antibody levels with respect to 25(OH)D levels but did not observe any significant relationships. Additional analyses included determining potential relationships between telomere length or anti-telomere antibody levels with specific disease characteristics, measures of disease damage, and disease activity at baseline and follow-up for SLE patients. Interestingly, anti-telomere antibody levels were significantly correlated with SLEDAI score, which was not observed in a subset of 48 SLE patients in a previous study. [20] Although largely Caucasian, the diverse genetic backgrounds of the 48 patients may account for the lack of a significant correlation in the previous study.

In conclusion, in a cohort of Gullah African American women we observed that SLE patients had significantly higher anti-telomere antibody levels and shorter telomeres than age- and gender-matched controls. We observed that anti-telomere antibody levels significantly correlated with SLEDAI score and may be an informative marker of disease in SLE worth further exploration. Although we did not observe any specific correlations between serum 25(OH)D levels and anti-telomere antibodies or telomere length at baseline in SLE patients, there was a significant correlation between telomere length and 25(OH)D level in all subjects (controls and patients) with the lowest 25(OH)D levels. There also was a trend toward patients with the lowest 25(OH)D levels having the shortest telomeres in the longitudinal analysis in which there was a significant increase in the average 25(OH)D levels. These studies support a relationship between vitamin D status and telomere length in patients with SLE. However, additional prospective follow-up of patients and controls with collection of samples over a longer period of time is needed to more precisely determine a possible relationship between telomere length and vitamin D status over time.
